# Low-Temperature Microbiology Meets the Global Challenges of Our Time

**DOI:** 10.3390/microorganisms11051217

**Published:** 2023-05-06

**Authors:** Amedea Perfumo, Angelina Lo Giudice

**Affiliations:** 1 Polar Terrestrial Environmental Systems, Alfred Wegener Institute (AWI) Helmholtz Centre for Polar and Marine Research, Telegrafenberg, 14473 Potsdam, Germany; 2 Department of Physics, Freie Universität Berlin, Arnimallee 14, 14195 Berlin, Germany; 3 Institute of Polar Sciences (CNR-ISP), National Research Council, Spianata S. Rainei 86, 98122 Messina, Italy

Low-temperature microbiology is intimately associated with the exploration of the polar regions, and research in recent decades has focused on characterizing the microbial biodiversity of the cryosphere [1] and elucidating the mechanisms of adaptation in extreme conditions [2]. Recently, a number of novel research domains have arisen at the interface with other disciplines, especially in relation to climate change, biotechnology, and public health. The aim of this Special Issue is to highlight the crucial role of psychrophilic microorganisms, both natural communities, strains, molecules and genes, in the context of these societal challenges.

Considering the potential impact on our society, asking how climate change affects environmental microbiomes, how they respond to it, and what the consequences are regarding the function of the ecosystem and the mechanisms that feed back to the climate is of the greatest relevance [3]. This is especially critical when the impact on cold ecosystems, which are particularly vulnerable to climate change compared to the rest of the biosphere, is considered. Polar oceans, for example, are currently undergoing dramatic transformations due to the climate, including a depletion of sea ice, water acidification, enlarged stratification, an adjusted current circulation, etc. All of these factors have severe implications for marine microbial communities [4]. In this Special Issue, Cordone and colleagues reveal that diverse bacterial phylotypes populate various water masses in Antarctic circumpolar currents, with the sea surface temperature and nutrient availability likely being the key drivers of alterations in the community composition. We can therefore expect the warming of the Southern Ocean to lead to species turnover, with a marked impact on the function of the ecosystem and on feedback to the Earth’s climate [5].

Connectivity amongst ecosystems plays a crucial role in climate change. The thawing of permafrost, for example, releases organic matter from the soil into lakes, streams and rivers, thus providing an input of carbon into freshwater ecosystems [6]. Similar dynamics occur in maritime Antarctic lakes, and with the terrestrial–aquatic connectivity expected to intensify within a warming climate, it is critical to understand the underlying metabolic potential and interactions that occur within the microbial communities inhabiting the lakes. In this Special Issue, Picazo and colleagues present a comparative analysis of the bacterioplankton communities that inhabit lakes in the maritime Antarctica. They evaluated the metabolic activities of these organisms regarding their carbon, nitrogen, sulfur, hydrogen and iron cycling, together with network associations; they observed marked distinctions between the function of the lake’s ecosystem and a clear relationship with the catchment. These findings have significant implications in the context of future climate scenarios, particularly with respect to regions in which stronger hydrological dynamics and connectivity within the catchment, together with an extended growing season, may notably accelerate the lake’s nutrient availability [7].

In addition to the general warming trend, climate change is elevating the frequency and magnitude of extreme climatic events, such as droughts, heat waves and floods, which can cause major fluctuations in the structure and function of ecosystems [8]. Our understanding of the response of microbial communities to abrupt environmental changes is, however, rather limited. In their contribution to this Special Issue, Bian and colleagues tackle the complications associated with the sudden freezing of nearshore seawater in mid-latitudes caused by cold surge outbreaks, and examine its impact on phytoplankton communities. The formation of sea ice, seldom occurring in the past in this non-polar region, promoted the growth of microalgae typical of polar regions such as *Bathycoccus* and *Micromonas*. Overall, the study offers the first insights into how microbial biodiversity and functionality alter with rapid and extreme climate events, which will become more recurrent in the near future [9].

In cold terrestrial ecosystems, it has been established that warming enhances the rate of microbial decomposition of soil organic matter, thus accelerating the rate of carbon flux into the atmosphere via greenhouse gas emission (carbon dioxide, methane). This likely triggers a positive feedback loop between climate and the carbon cycle [10]. Poyntner and colleagues contributed to this Special Issue with a study on the degradation of lignin derivatives (lignin sulfonic acid, catechol and phenol) by bacteria isolated from Alpine forest soils. By employing PCR in combination with culture-based screening in order to detect degradative enzymes, they assessed the biodegradation potential of a large number of cold-adapted bacteria, including *Pseudomonas*, *Collimonas* and *Rhodococcus* [11]. Works of this kind enable us to adeptly estimate the contribution of microbes to carbon fluxes in nature, but also their high biotechnological impact.

The development of a circular bioeconomy that is based on sustainable bioprocessing and employs recyclable material in order to manufacture high-value products is another grand challenge posed to our society. Microbial enzymes are promising biocatalysts that can be applied in many industrial production processes. In particular, the valorization of lignocellulosic biomass via lignin biodegradation is attracting significant attention, and intense research is being directed towards the discovery and implementation of microbial enzymatic activities [12].

In general, psychrophilic microorganisms have high biotechnological potential owing to their biosustainability and performance under harsh conditions, and cold-active enzymes and biosurfactants have, so far, been the primary target of research [13]. In this Special Issue, Rizvi and colleagues discussed the application of cold-active phosphate solubilizing bacteria as environmentally friendly biofertilizers in order to improve crop production in cold regions. With the rapid inflation of food demand worldwide, extending agricultural systems to cold areas would be highly favorable. Plant beneficial psychrophilic bacteria may be used in this respect in order to support plant growth in cold soils by increasing nutrient availability (e.g., phosphorous) [14].

Another emerging topic that is not separately addressed in this Special Issue, but it is pressing and alarming, relates to the potential impact of the melting of the cryosphere on public health. The thawing of permafrost, for example, may unearth biological, chemical and radioactive materials that have been buried and frozen underground until now. If they re-enter the environment, they have the potential to disrupt ecosystems and endanger human life. For example, various viruses and bacteria have been revived from ancient permafrost, posing the question of whether outbreaks of human diseases (but also animal and plant), caused by the revival of ancient unknown pathogens, are possible [15]. The pollution of the cryosphere with microplastics is also of significant environmental concern, with serious implications for ecosystem function and services, as well as public health. Several studies have demonstrated the presence of these contaminants in glaciers in the Arctic, Antarctica and the Third Pole, highlighting the possibility that, via the melting of snow and ice, they can be transported via freshwater streams and enter the food chain, becoming a potentially serious health threat to humans [16].

Overall, the low-temperature microbiology of the 21st century is significantly broadening its scientific scope and perspectives, providing critical contributions to the consideration of various societal challenges, ranging from global climate change to a sustainable bioeconomy and public health, and reaching out to the attention of policy makers and the general public. With climate-related issues becoming an urgent priority, it is anticipated that scientific and societal interest towards cryo-environments and cryophiles will strongly converge in the near future.
